# Development and clinical application of a novel CRISPR-Cas12a based assay for the detection of African swine fever virus

**DOI:** 10.1186/s12866-020-01966-6

**Published:** 2020-09-14

**Authors:** Xiaoying Wang, Sheng He, Na Zhao, Xiaohong Liu, Yongchang Cao, Guihong Zhang, Gang Wang, Chunhe Guo

**Affiliations:** 1 State Key Laboratory of Biocontrol, School of Life Sciences, Sun Yat-sen University, North Third Road, Guangzhou Higher Education Mega Center, Guangzhou, Guangdong 510006 PR China; 2 Precise Genome Engineering Center, School of Life Sciences, Guangzhou University, Guangzhou, Guangdong 510006 PR China; 3 College of Veterinary Medicine, South China Agricultural University, Guangzhou, Guangdong 510642 PR China; 4 African Swine Fever Regional Laboratory of China (Guangzhou), Guangzhou, Guangdong 510642 PR China

**Keywords:** African swine fever; CRISPR-Cas12a; nucleic acid detection; diagnosis; qPCR

## Abstract

**Background:**

As no treatment or effective vaccine for African swine fever virus (ASFV) is currently available, a rapid, highly sensitive diagnostic is urgently needed to curb the spread of ASFV.

**Results:**

Here we designed a novel CRISPR-Cas12a based assay for ASFV detection. To detect different ASFV genotypes, 19 crRNAs were designed to target the conserved p72 gene in ASFV, and several crRNAs with high activity were identified that could be used as alternatives in the event of new ASFV variants. The results showed that the sensitivity of the CRISPR-Cas12a based assay is about ten times higher than either the commercial quantitative PCR (qPCR) kit or the OIE-recommended qPCR. CRISPR-Cas12a based assay could also detect ASFV specifically without cross-reactivity with other important viruses in pigs and various virus genotypes. We also found that longer incubation times increased the detection limits, which could be applied to improve assay outcomes in the detection of weakly positive samples and new ASFV variants. In addition, both the CRISPR-Cas12a based assay and commercial qPCR showed very good consistency.

**Conclusions:**

In summary, the CRISPR-Cas12a based assay offers a feasible approach and a new diagnostic technique for the diagnosis of ASFV, particularly in resource-poor settings.

## Background

African swine fever (ASF) is one of the most important zoonotic diseases that can lead to high mortality in domestic pigs, leading to huge economic losses [1, 2]. ASF is caused by African swine fever virus (ASFV), a large, enveloped, double-stranded DNA virus that is the sole member of the Asfarviridae family [3]. The viral genome comprises between 170 and 190 kilobases that encode more than 150 ORFs and approximately 165 viral proteins [4]. The viral capsid protein p72, encoded by the *B646L* gene, is highly conserved and well-characterized [5, 6], making it a widely used target for both nucleic acid detection and phylogenetic analysis [7, 8].

African swine fever was first documented in Kenya in 1921 and later discovered in most countries in Africa, and it was introduced into Europe in the middle of the last century [1, 9]. In 2007, an outbreak of this disease was reported in the Caucasus region, including Georgia, Armenia, Azerbaijan, and Russia [9] and since then has been isolated in neighboring countries (Ukraine in 2012, Belarus in 2013, Lithuania, Poland, Latvia and Estonia in 2014, the Czech Republic in 2017) [1, 10]. It eventually travelled to China, where the first serious outbreak was reported in 2018 and has since spread to many of the country’s provinces [3, 11]. These outbreaks were only brought under control following animal quarantine and eradication, which came at a very high economic cost.

Since there is no effective vaccine or antiviral drug currently available, early detection and surveillance of this disease are very important for preventing and controlling outbreaks and reducing economic losses. The diagnosis of ASFV normally relies on both viral genome and antibody detection. When using viral antibody detection, most serological detection assays rely on the ELISA format as it is simple, high throughput, and cost-effective [12, 13]. However, antibody induction takes a comparatively long time following viral infection, and in many cases, this means that the animals may die before the antibody reaches detectable levels [12]. In the case of detection using the viral genome, both conventional and quantitative PCR are recommended by World Organization for Animal Health as the gold standard for detection as a result of their high sensitivity and specificity [12, 14]. The main drawback of either technique is the requirement of laboratory facilities equipped with expensive machinery.

The clustered regularly interspaced short palindromic repeats (CRISPR)-associated endonuclease Cas from prokaryotic immune systems has been developed and employed as a robust genome-editing tool. Recently, several endonucleases (Cas12a/b, Cas13a/b, and Cas14) from the CRISPR-Cas system were found to possess collateral cleavage activity that can degrade single-stranded DNA or RNA nonspecifically upon binding to their target site. This ability can be used to detect pathogens when a short reporter sequence is also added to the cells [15–18]. This discovery has led to the emergence of many CRISPR-Cas based virus detection protocols. This technology could be an ideal tool in diagnostics as a result of its excellent performance in several important areas including high sensitivity, specificity, and accuracy as well as the fact that this technology is rapid and easy to use [15, 17–21].

To overcome the limitations and improve the existing tools for ASFV diagnostics, we explored the application of novel CRISPR-Cas12a based viral detection assay in the detection of ASFV. Here, we report findings that strongly suggest that this approach could be a novel ASFV diagnostic assay.

## Results

### crRNA design and development of a CRISPR-Cas12a based ASFV detection assay

**Fig. 1:**
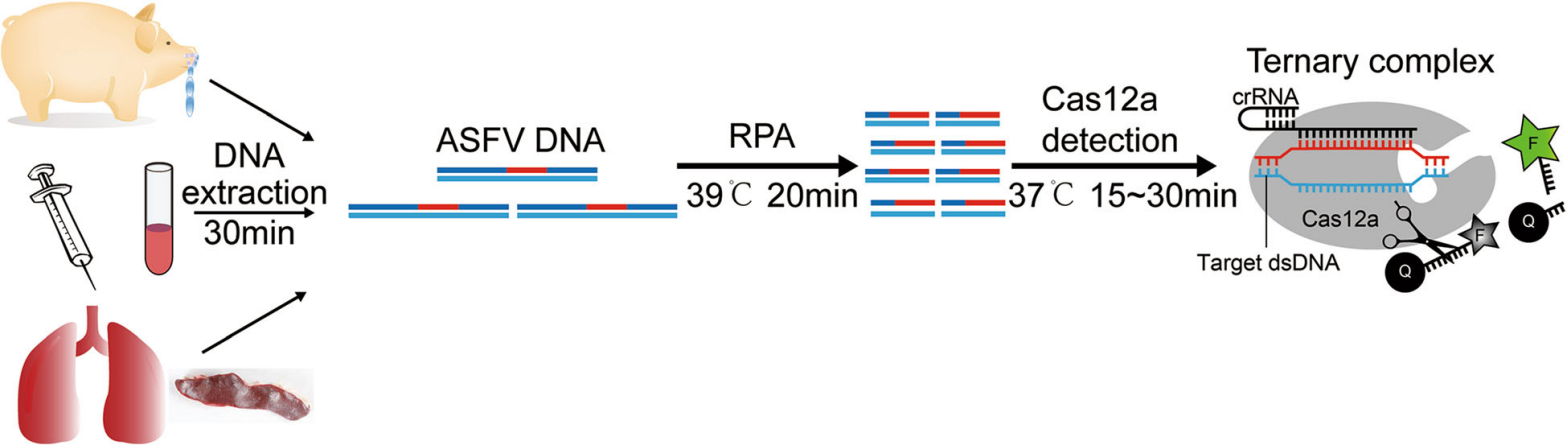
Schematic of the CRISPR-Cas12a based assay for ASFV detection. Viral nucleic acids from different sample types were extracted, and the p72 target region amplified using RPA at 39 °C for 20 min. The amplicon was then mixed with Cas12a/crRNA, and a ternary complex formed when the target region was present. Finally binding to the target site resulted in the cleavage of the reporter ssDNA producing a signal. The image shown in Fig. 1 was made by us and belongs to us

A schematic for the application of CRISPR-Cas12a in ASFV detection is shown in Fig. 1. Viral nucleic acids were extracted, and the p72 target region amplified, using isothermal RPA. This amplicon was then mixed with Cas12a/crRNA, and a ternary complex formed when the target region was present in the DNA. Binding to the target site resulted in the cleavage of the reporter ssDNA constructs producing a signal [22].

The p72 gene (*B646L*) was chosen as the crRNA target site for these assays because of its high degree of conservation and its wide application as a diagnostic target. Twenty-four genotypes of ASFV have been characterized using the partial p72 gene sequence. To enable this assay to detect various known ASFV genotypes, 38 p72 gene sequences were obtained from GenBank and aligned to identify conserved regions. A total of 19 “universal” crRNAs were designed to target these conserved regions (Table S1). Universal RPA primers that amplify all viral genotypes were also designed (Table S2).

**Fig. 2:**
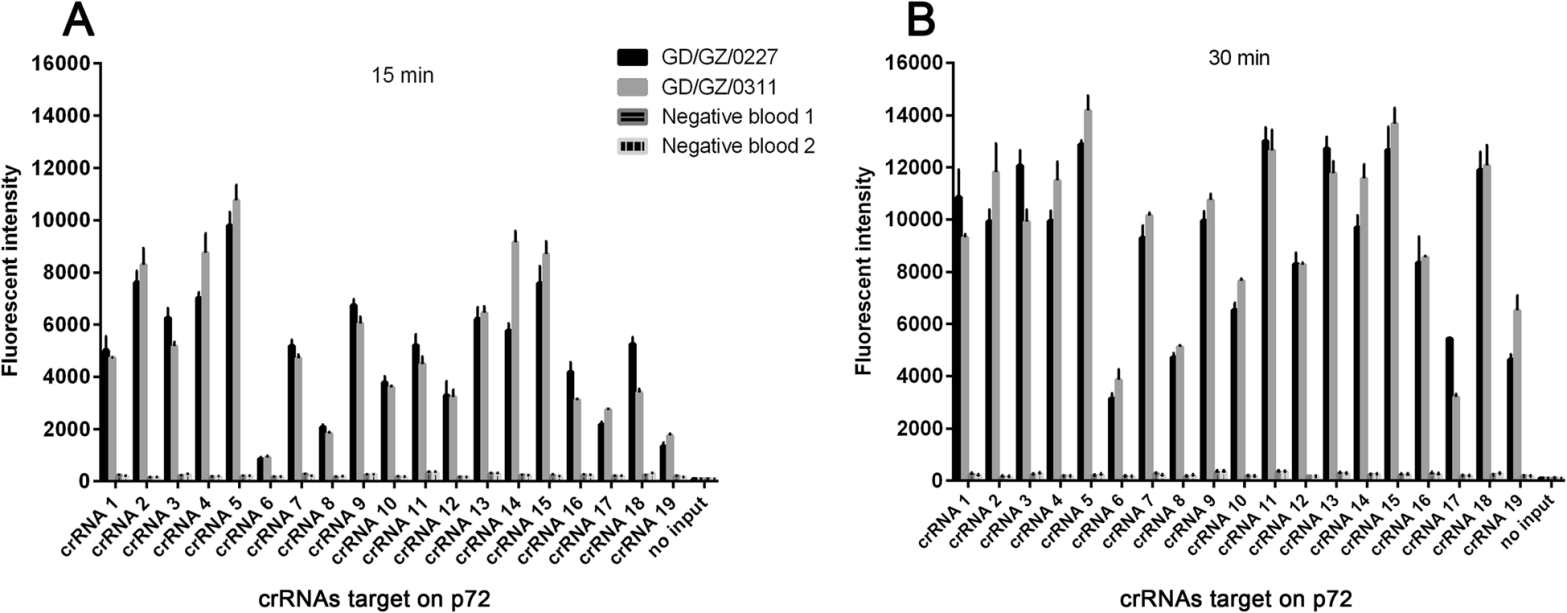
Screening for highly active crRNAs. A total of 19 crRNAs targeting the ASFV p72 gene were designed, these should work as a “universal” crRNA set for the detection of ASFV. Two ASFV positive blood (GD/GZ/0227, GD/GZ/0311) and two negative blood were used to test crRNA activity for 15 (**a**) and 30 (**b**) min. Fluorescence signal reflects the activation of individual crRNA, *n =* 3 technical replicates; bars represent mean ± SD

To optimize the sensitivity, the efficiency of all 19 crRNAs was evaluated. We used two ASFV positive blood (GD/GZ/0227 and GD/GZ/0311) collected from a pig farm with an outbreak of ASFV for this evaluation. The viral loads of these two ASFV positive samples are 7.3 × 10^4^ and 8.6 × 10^4^ copies/μl, respectively. Two ASFV negative blood samples from an ASFV-negative pig farm in Jiangxi province of China were also tested parallelly to compare the crRNAs activity between positive and negative samples. Variable performance efficiency for each crRNA was observed (Fig. 2a and b), with crRNA5 showing the highest activity. This crRNA was selected to perform the rest of the evaluations.

### Evaluation of the sensitivity and specificity of the CRISPR-Cas12a detection assay

**Fig. 3:**
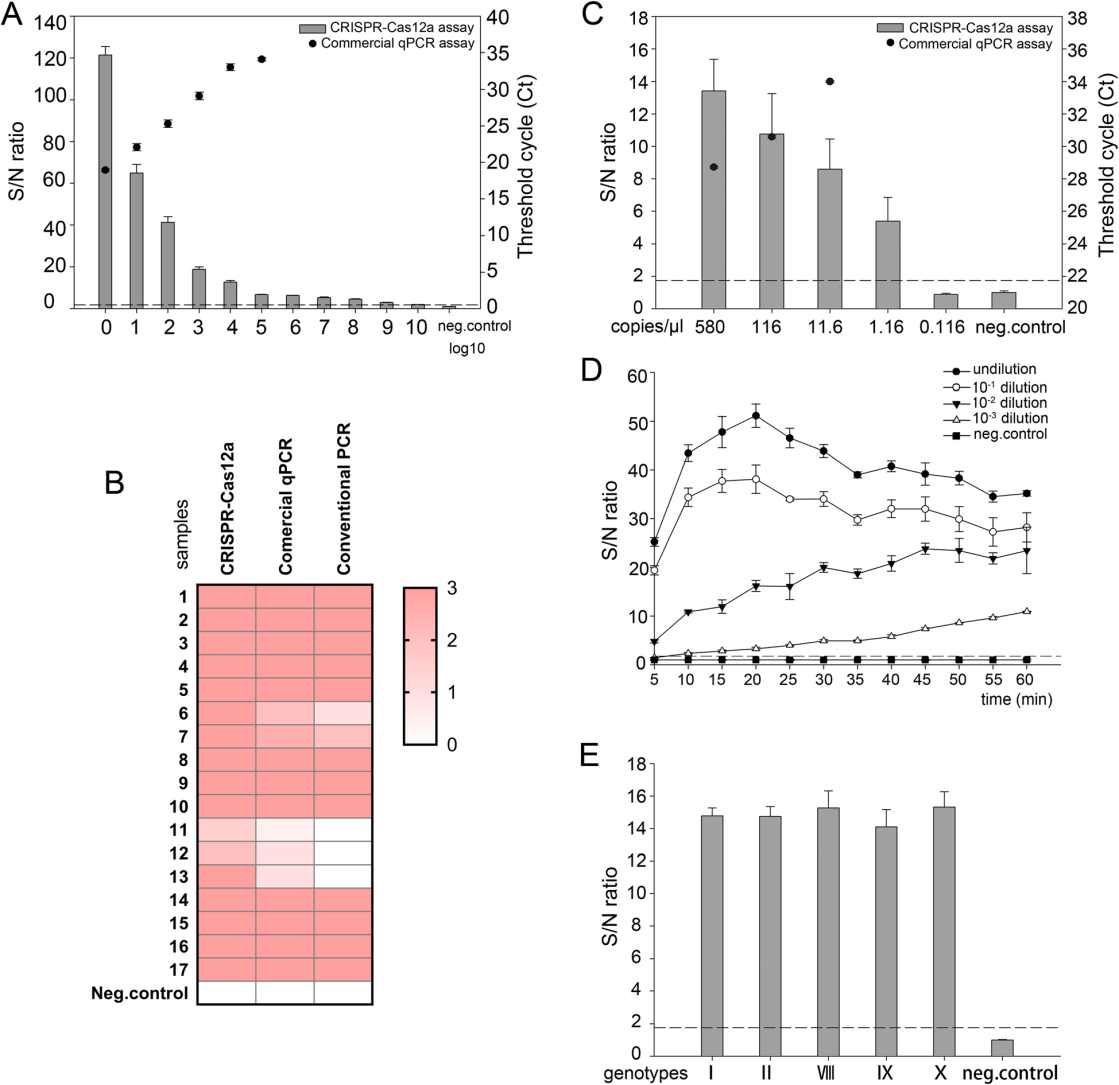
Sensitivity of CRISPR-Cas12a based assay in the detection of ASFV. **a** A qPCR positive blood sample (GD/GZ/0311) was subjected to serial (log10) dilution and examined by both the CRISPR-Cas12a and the commercial qPCR assay. Columns – CRISPR-Cas12a based assay; black dot – commercial qPCR assay. The dashed line represents the detection threshold for the virus. **b** A total of 17 qPCR positive blood were used for comparison using all three nucleic acid detection methods. Heatmap results represent the signal intensity of individual samples. **c** ASFV *B646L* Gene Plasmid Reference Material (5.8 × 10^3^ copies/μl) was subjected to serial dilution and examined by the CRISPR-Cas12a and the commercial qPCR. Columns – CRISPR-Cas12a based assay; black dot – commercial qPCR assay. Dashed line represents the detection threshold for the virus. **d** The panel shows that serial dilution of the positive sample (GD/GZ/0311) remained detectable over a 1 h time course with S/N ratio (fluorescence) measurements every 5 min. **e** The p72 genes of various ASFV genotypes I, II, VIII, IX, and X were synthesized and used to assess whether the CRISPR-Cas12a assay has the ability to test different ASFV genotypes. The dashed line represents the detection threshold for the virus. *n =* 3 technical replicates, bars represent mean ± SD

First, we compared the sensitivity of the CRISPR-Cas12a assay with the commercial qPCR kit (Beijing Anheal Laboratories, China) using a 1:10 serial dilution of positive sample (GD/GZ/0311, qPCR [Ct] = 17). As shown in Fig. 3a, the sample diluted to 10^− 8^ still could be definitely detected by the CRISPR-Cas12a assay, while the limit of detection for the commercial qPCR kit was 10^− 5^. Seventeen qPCR-positive clinical samples were then tested to validate the sensitivity of the CRISPR-Cas12a detection assay, and conventional PCR was also used as confirmation of our findings. All 17 samples were confirmed positive by the CRISPR-Cas12a assay, while three weakly positive samples could not be detected using conventional PCR (Fig. 3b). In addition, five samples had superior signal strength in the CRISPR-Cas12a assay when compared to their results using the commercial qPCR kit. To further quantify the sensitivity of these two methods, ASFV *B646L* Gene Plasmid Reference Material (number: GBW(E)091034, 5.8 × 10^3^ copies/μl), developed by China Animal Disease Control Center, was used. As shown in Fig. 3c, the limit of detection of the commercial qPCR kit was 11.6 copies/μl. However, the limit of the CRISPR-Cas12a was as low as 1.16 copies/μl. Since the Office International Des Epizooties (OIE)-recommended qPCR was recognized as the gold standard, we also compared the sensitivity and specificity of the commercial qPCR kit with the OIE-recommended qPCR [23]. As expected, the detection limit of the commercial qPCR was comparable to the OIE-recommended qPCR (Table S3). Consistently, similar to the OIE-recommended qPCR, the commercial qPCR showed no cross-reactivity with other swine viruses including PRRSV, CSFV, PCV2, PRV, PEDV, TGEV, JEV, and PPV (Table S4). Taken together, these data show that the CRISPR-Cas12a detection assay has a significantly higher sensitivity (about ten times) than either the commercial qPCR kit or the OIE-recommended qPCR.

In addition, longer incubation times were used to evaluate whether assay sensitivity can be improved in weakly positive samples. We used serial dilutions of the sample (GD/GZ/0311) to mimic a weakly positive blood sample. In this assay, the incubation time for Cas12a/crRNA was increased to 60 min. The results showed that fluorescence values for the 10^− 3^ dilution were increased with longer incubation times but the values in the negative sample remained unchanged (Fig. 3d). To test whether the CRISPR-Cas12a is capable of detecting various ASFV genotypes, we synthesized the conserved p72 gene of genotypes I (GenBank No: FN557520), II (GenBank No: MK128995), VIII (GenBank No: AY261361), IX (GenBank No: MH025920), and X (GenBank No: KM111294) (Sangon Biotech, China). As shown in Fig. 3e, as expected, the S/N ratios of all genotypes were significantly above the cut-off values, indicating that the CRISPR-Cas12a can test different ASFV genotypes.

**Fig. 4:**
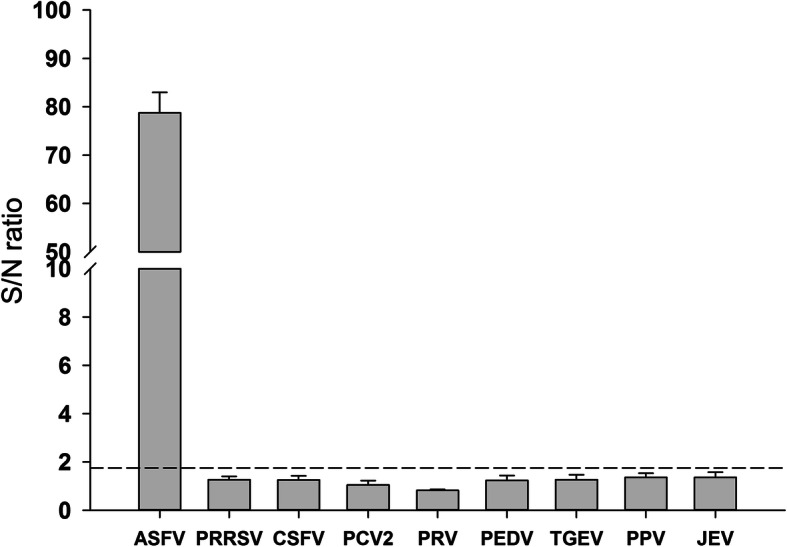
Specificity of the CRISPR-Cas12a based assay for ASFV detection. Specificity test results compared specificity of the CRISPR-Cas12a based assay in ASFV against its activity in other important pig viruses. *n =* 3 technical replicates, bars represent mean ± SD. The dashed line means detection threshold for each virus

Specificity of CRISPR-Cas12a assay was evaluated using blood (GD/GZ/0311) and genomic material from other swine viruses including PRRSV, CSFV, PCV2, PRV, PEDV, TGEV, JEV, and PPV. As expected, no cross-reactivity with other viruses was observed (Fig. 4). These results demonstrate that CRISPR-Cas12a assay is specific and sensitive and thus could be used in the specific detection of various ASFV genotypes.

### Evaluating consistency between the CRISPR-Cas12a assay and the commercial qPCR kit

**Fig. 5:**
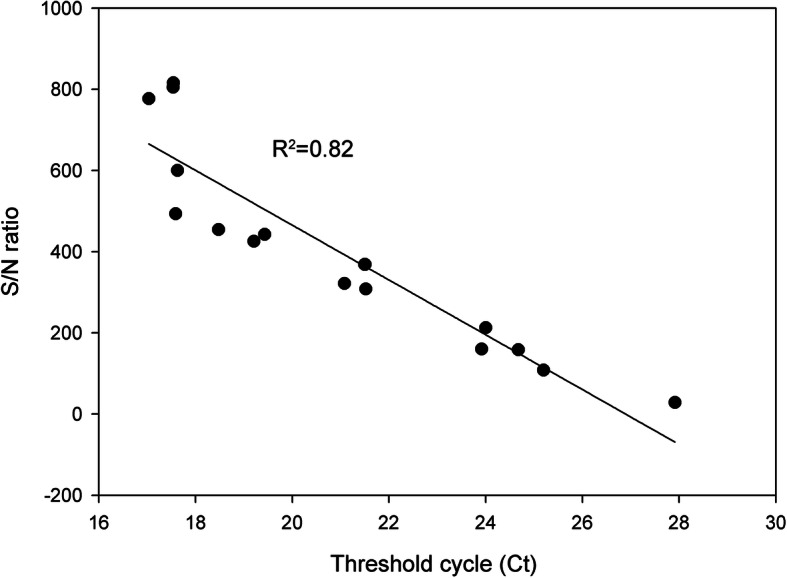
Consistency in evaluation between CRISPR-Cas12a and commercial qPCR assays during ASFV detection. A total of 17 qPCR ASFV-positive blood were also confirmed positive by CRISPR-Cas12a assay. An inverse linear relationship (R^2^ = 0.82) exists between the cycle threshold and S/N ratio

The 17 qPCR-positive clinical samples mentioned above were also used to evaluate the consistency in positive sample identification between CRISPR-Cas12a and the commercial qPCR kit. As expected, there was high concordance (R^2^ = 0.82) between the two methods (Fig. 5), which indicates good ASFV diagnostic performance for this novel assay.

### Detection of ASFV nucleic acids in porcine samples from the field

**Fig. 6:**
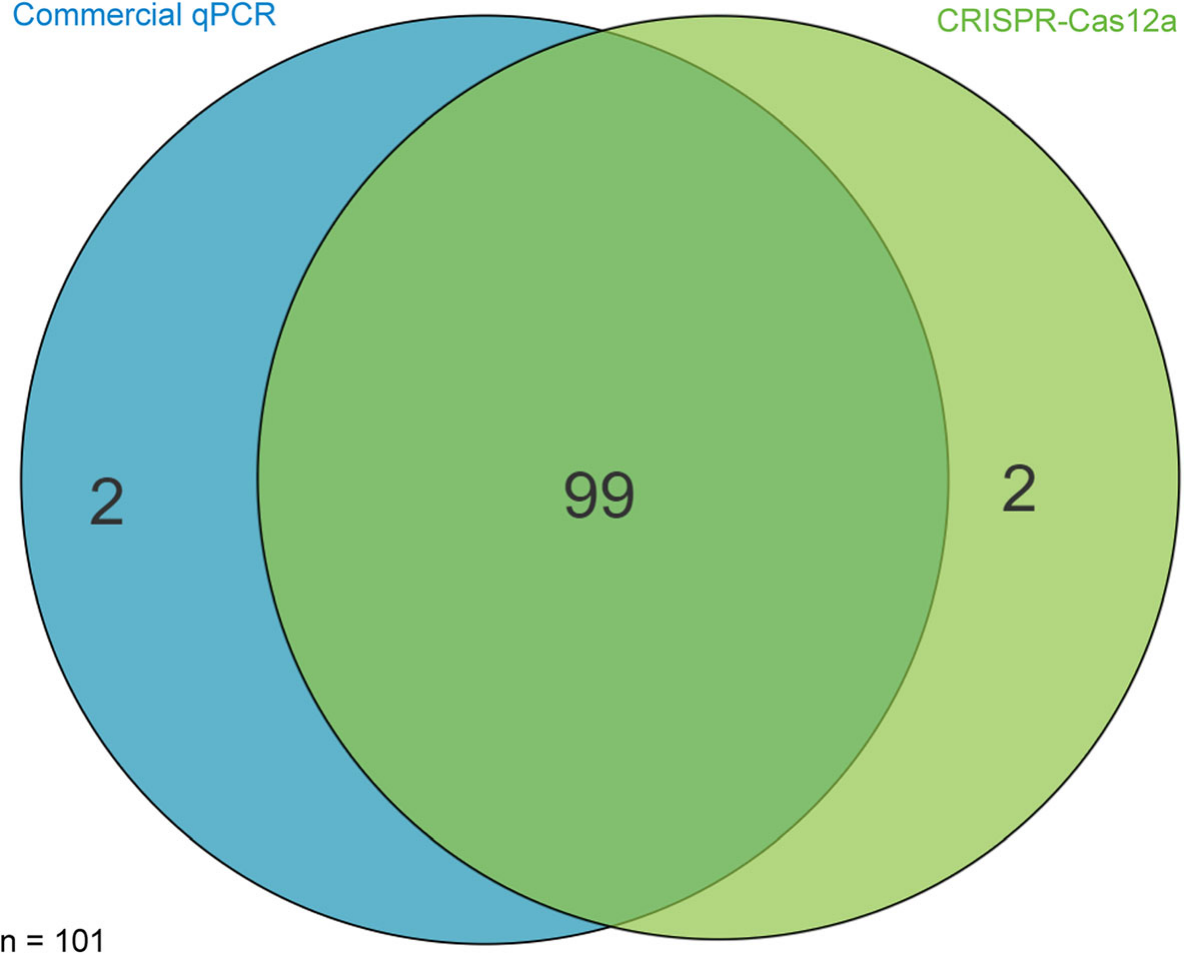
Venn diagram of detection of ASFV by commercial qPCR (blue circle) and CRISPR-Cas12a (green cycle) assays using porcine blood from the field (*n* = 101). Only two samples produced incongruent results in both tests

Detection of ASFV in a collection of 101 porcine field samples including blood, oral fluid and spleen was compared for the CRISPR-Cas12a and the commercial qPCR assays. As shown in Tables 1, 18 out of 67 blood samples and 13 out of 18 oral fluid samples tested positive using the commercial qPCR assay. While one more sample was positive in the blood and oral fluid groups respectively when they were evaluated using the CRISPR-Cas12a assay. This may be because the sensitivity of CRISPR-Cas12a detection is higher than that of the commercial qPCR kit. In addition, 11 out of 16 spleen samples tested positive in both assays. We used a Venn diagram to depict the results of the CRISPR-Cas12a and commercial qPCR assays. As shown in Fig. 6, a high degree of agreement between these two assays is evident, which supports the application of this novel diagnostic assay in the detection of ASFV.

**Table 1:** Parallel detection data from both the CRISPR-Cas12a and commercial qPCR assays in clinical samples

Sample types	Nucleic acid detection assays	
	CRISPR-Cas12a assay	Commercial qPCR
Blood	19/67^a^	18/67
Oral fluid	14/18	13/18
Spleen	11/16	11/16

^a^ number of positive/total samples

## Discussion

Chinese pig farming accounts for 60% of the global pig production industry. The first ASF case in China was reported in August 2018 and the virus has rapidly spread throughout the whole country, causing serious losses in the Chinese swine industry. As no effective vaccine or treatment is available, ASFV can lead to pig death very quickly even without clinical symptoms and antibody response, this means that early detection and surveillance of this disease is vital to prevent its outbreak and spread. Therefore, developing a method for ASFV detection using minimal equipment is urgently needed. To the best of our knowledge, this is the first report that aims to employ CRISPR-Cas12a based assay for the detection of ASFV.

The Cas12a, Cas13a systems were developed and have the potential to become a promising onsite detection method. Cas12a-based assay is especially suitable for DNA virus detection because the product of target site amplification can react with Cas12a directly without having to perform transcription procedure. Furthermore, Cas12a is a more cost-effective assay than Cas13a: obviating the need for T7 RNA polymerase and related regents as well as the more expensive di-labelled RNA reporter [24]. Additionally, the CRISPR/Cas assay can be run in approximately 30–40 min and visualized on a lateral flow strip. Therefore, Cas12a is a promising tool in the detection of ASFV.

Sensitivity plays a decisive role in developing an alternative diagnostic assay for ASFV. A previous study showed that RPA with lateral flow dipstick is developed for the on-site detection of ASFV [25], but the limit of detection of this method is 150 copies per reaction, which is much lower than the present gold standard test qPCR. So the clinical application of this method is limited. Previous studies also showed that CRISPR system can detect ASFV [26, 27]. However, they did not compare the sensitivity of CRISPR with qPCR, the most sensitive method in the detection of ASFV until now, indicating that further research is needed in the sensitivity analysis of the CRISPR in ASFV detection. Furthermore, to date twenty-four genotypes of ASFV have been characterized based on the p72 gene sequence which is used to design crRNA, but the previous research [26, 27] tested only genotype II ASFV that emerged in China using CRISPR system, which means that whether the CRISPR can detect other genotypes is unknown. In this study, to accurately quantify the limit of detection of the CRISPR-Cas12a, we used a ASFV B646L Gene Plasmid Reference Material (number: GBW(E)091034) rather than a synthetic ASFV DNA [26, 27]. We found that the CRISPR-Cas12a reached a sensitivity level of 1.16 copies/μl, which has a much higher sensitivity (about ten times) than either the commercial qPCR assay or the OIE-recommended qPCR (Fig. 3c, Table S3). We also tested the sensitivity of RPA in the detection of ASFV, and the detection limit of RPA was 116 copies/μl (data not shown), which was about one hundred times lower than that of CRISPR-Cas12a. In addition, we also showed that the CRISPR-Cas12a could test different ASFV genotypes (Fig. 3e). These data indicate that the CRISPR-Cas12a is a promising tool for the rapid detection of ASFV at the early stage of infection before clinical signs emerged.

In the ASFV detection using the clinical samples from the field, the results of the CRISPR-Cas12a and the commercial qPCR showed a good consistency (Figs. 5 and 6, Table 1), but the test process of the CRISPR-Cas12a requires less time than the commercial qPCR. The CRISPR-Cas12a also showed highly specific activity without cross-reactivity, which may be due to the combination of RPA specific amplification and Cas12a/crRNA sequence identification. This study highlights CRISPR-Cas12a based assay as a promising diagnostic technique for ASFV detection in clinical application.

To optimize the detection sensitivity of the CRISPR-Cas12a, we identified crRNAs with a higher affinity for the target sequence, which can be used to detect various known ASFV variants. Several crRNAs showed high affinity meaning that they could be used as an alternative in case a new ASFV variant emerges. Because the mismatch between crRNA and the target site on ASFV DNA genome could affect the affinity, the crRNAs were carefully designed to target only highly conserved regions of viral p72 gene avoiding possible mutations in the target site to the utmost extent. Therefore, crRNA activity and conservation of the crRNA target site are equally important in the global detection of ASFV when using a CRISPR-Cas12a based assay.

Genome-based diagnosis of viral infections is significantly challenged by high mutation rates in their selected target sites. Mutations at the crRNA target site could affect the activity of this assay resulting in reduced sensitivity [28]. Here, we found that the S/N ratio of weakly positive samples became stronger when extending incubation time, which makes CRISPR-Cas12a as a promising alternative for the detection of ASFV. Furthermore, we confirmed that the CRISPR-Cas12a assay can test different virus genotypes (Fig. 3e), suggesting that this approach also could be used to detect new ASFV variants to some extent, which might make a vital contribution to the control of ASFV.

## Conclusions

In conclusion, we have developed a new and feasible CRISPR-Cas12a based assay for the detection and diagnosis of ASFV. This assay is characterized by its high sensitivity, ease of use, short protocol time and requires minimal equipment making it an ideal candidate for the rapid detection and surveillance of ASFV in China. Further studies are needed to evaluate its potential for clinical application in the future.

## Methods

### Sample collection and viruses

In this study, we collected 101 blood, tissue, and oral fluid samples from pig farms located in the Guangdong province of China. Porcine reproductive and respiratory syndrome virus (PRRSV), pseudorabies virus (PRV), porcine circovirus type 2 (PCV2), classical swine fever virus (CSFV), porcine epidemic diarrhea virus (PEDV), porcine transmissible gastroenteritis virus (TGEV), Japanese encephalitis virus (JEV), and porcine parvovirus (PPV) were available in our laboratory.

### Viral genome extraction and crRNA preparation

Viral nucleic acids from the blood samples were extracted using the RaPure Viral RNA/DNA Kit (Magen, China) according to the manufacturer’s instructions. Probe-based qPCR analysis was carried out using the AFD9600 Real-time System (AGS BioTech Co., China). Viral nucleic acids were eluted in 20 μl of nuclease-free water and stored at − 80 °C until use. For crRNA preparation, based on the conserved p72 sequence of ASFV, crRNAs were designed (Table S1) and cloned with a T7 promoter. The transcription templates were prepared by annealing the synthesized oligonucleotides. Then, crRNAs were transcribed and purified using a T7 High Yield Transcription Kit (Thermo Fisher Scientific) and an RNA Clean & Concentrator™-5 Kit (Zymo Research) respectively, each according to the manufacturer’s instructions. Finally, crRNAs were quantified using the Nanodrop 2000C (Thermo Fisher Scientific) and stored at − 80 °C.

### CRISPR-Cas12a assay

The detection of ASFV by probe-based qPCR has been described previously [29–31]. In terms of African swine fever virus (ASFV), qPCR is the gold standard, which we used as a reference for our CRISPR-Cas12a assay. LbCas12a (New England Biolabs) was used for this assay. As described in the DETECTR method, our CRISPR-Cas12a assay also uses recombinase polymerase amplification (RPA). For RPA reactions, the TwistAmp Basic kit (TwistDx) was used according to the manufacturer’s instructions (https://www.twistdx.co.uk/en). Briefly, 50 μl reactions containing 2.5 μl ASFV DNA, 0.48 μM forward and reverse primers, 1× rehydration buffer, 14 mM magnesium acetate and RPA mix were incubated at 39 °C for 20 min. CRISPR-Cas12a detection was performed as described previously with minor modifications. The reaction volume was a total of 50 μl, with 20 nM crRNA, 115 nM single-stranded DNA fluorophore quencher - labeled reporter (Table S1), 30 nM LbCas12a, 1 μl RPA amplification products, 2 μl RNase inhibitor (New England Biolabs), and 1 × LbCas12a Buffer (New England Biolabs). The reactions were incubated in a temperature-controlled water bath for 15 min at 37 °C. Fluorescence emission was excited at 485 nm and detected at 535 nm using a fluorescent microplate reader (BioTek), and reactions without target DNA were used to establish the background.

### **Sensitivity of the CRISPR-Cas12a detection assay**

To determine the sensitivity of the CRISPR-Cas12a assay in the detection of ASFV, we serially diluted an ASFV-positive blood sample using a 10-fold gradient. Genomic extraction was performed using the RaPure Viral RNA/DNA Kit (Magen). CRISPR-Cas12a and probe-based qPCR detection were performed. When the genomic DNA of diluted samples were tested using CRISPR-Cas12a, we evaluated the exposure times for each dilution to allow for optimization of the protocol.

### **Specificity of the CRISPR-Cas12a detection assay**

The specificity of the ASFV CRISPR-Cas12a assay was evaluated by using it to detect various important pathogenic viruses of swine including ASFV, PRRSV, PRV, CSFV, PCV2, PEDV, TGEV, PPV, and JEV. Distilled water served as a negative control. Viral nucleic acids were extracted and the targets were amplified using the ASFV RPA primers described above. CRISPR-Cas12a complexes were then administered and the assay performed.

### Determination of cut-off values for CRISPR-Cas12a assay

A total of 47 blood samples, originated from an ASFV-negative pig farm in Jiangxi province of China, were used to determine the cut-off values in the CRISPR-Cas12a assay. All these samples tested negative for ASFV using the commercial qPCR kit (Beijing Anheal Laboratories, China). Mean extinctions and standard deviations (SD) were calculated. Blood_neg01 was chosen as a standard negative control. This control was used as the standard in each assay to determine threshold values and to calculate S/N ratios. The S/N mean value for all 47 negative blood plus 3 SD was chosen as the endpoint for all serological assays (Table 2). Results were calculated and expressed in S/N units: S/N ratio = sample test (fluorescent intensity)/negative test (fluorescent intensity).

**Table 2:** Determination of cut-off values for the CRISPR-Cas12a based assay during the time course study

S/N ratio	Time point (min)													
	0	5	10	15	20	25	30	35	40	45	50	55	60	90
Mean	0.96	1.03	1.03	1.03	1.05	1.07	1.06	1.14	1.10	1.13	1.11	1.08	1.09	1.07
SD	0.21	0.21	0.23	0.24	0.26	0.25	0.26	0.26	0.28	0.29	0.29	0.30	0.30	0.32
mean + 3SD	1.58	1.67	1.73	1.75	1.82	1.81	1.85	1.92	1.93	2.01	1.98	1.98	1.98	2.03

### Conventional PCR and qPCR assays for detection of ASFV

Amplification of p72 gene from ASFV by conventional PCR was performed using the TaKaRa ExTaq kit (TaKaRa, Japan). Each 20 μl amplification reaction contained primers (Table S2) at a final concentration of 300 nM and varied concentrations of the template. The PCR conditions were as follows: 95 °C for 10 min followed by 30 cycles at 95 °C for 30 s, 51 °C for 30 s, and 72 °C for 40 s with a final extension at 72 °C for 10 min. Amplified PCR products were visualized using 1% agarose gels. The qPCR assay was performed using a commercial kit (Beijing Anheal Laboratories, China) and completed according to the manufacturer’s instructions. Briefly, each reaction was a total of 10 μl, 5 μl qPCR Mix, 0.4 μl ASFV sense and 0.4 μl ASFV anti-sense primers, 0.4 μl TaqMan probe, 1.8 μl nuclease-free water, and 2 μl DNA template. The cycling protocol was as follows: 1 cycle of 95 °C for 9 min followed by 40 cycles made up of denaturation for 15 s at 95 °C and annealing for 45 s at 60 °C. The results were analyzed using cycle threshold values.

## Supplementary information

**Additional file 1: Table S1.** List of crRNA spacer sequences and DNA reporters used in this study. **Table S2.** List of RPA and conventional PCR primer sequences used in this study. **Table S3.** The sensitivity comparison between commercial qPCR kit and OIE-recommended qPCR. **Table S4.** The specificity comparison between commercial qPCR kit and OIE-recommended qPCR

## Data Availability

The datasets generated and/or analysed during the current study are not publicly available due to ongoing research on this datasets since this study is a part of a thesis but are available from the corresponding author on reasonable request.

## Abbreviations

ASFV: African swine fever virus
PRV: Pseudorabies virus
PCV2: Porcine circovirus type 2
CSFV: Classical swine fever virus
PEDV: Porcine epidemic diarrhea virus
TGEV: Porcine transmissible gastroenteritis virus
JEV: Japanese encephalitis virus
PPV: Porcine parvovirus
PRRSV: Porcine reproductive and respiratory syndrome virus
Ct: Cycle threshold
qPCR: Quantitative polymerase chain reaction

## References

[1] Galindo I, Alonso C. African swine fever virus: a review. Viruses. 2017;9(5). 10.3390/v9050103. 10.3390/v9050103 PMC5454416 28489063

[2] Andraud M, Halasa T, Boklund A, Rose N (2019). Threat to the French swine industry of African swine fever: surveillance, spread, and control perspectives. Front Vet Sci.

[3] Zhao D, Liu R, Zhang X, Li F, Wang J, Zhang J (2019). Replication and virulence in pigs of the first African swine fever virus isolated in China. Emerg Microbes Infect.

[4] Wen X, He X, Zhang X, Liu L, Guan Y, Zhang Y (2019). Genome sequences derived from pig and dried blood pig feed samples provide important insights into the transmission of African swine fever virus in China in 2018. Emerg Microbes Infect.

[5] Bao J, Wang Q, Lin P, Liu C, Li L, Wu X (2019). Genome comparison of African swine fever virus China/2018/AnhuiXCGQ strain and related European p72 genotype II strains. Transbound Emerg Dis.

[6] Olasz F, Meszaros I, Marton S, Kajan GL, Tamas V, Locsmandi G, et al. A simple method for sample preparation to facilitate efficient whole-genome sequencing of African swine fever virus. Viruses. 2019;11(12). 10.3390/v11121129. 10.3390/v11121129 PMC6950082 31817647

[7] Gilliaux G, Garigliany M, Licoppe A, Paternostre J, Lesenfants C, Linden A, et al. Newly emerged African swine fever virus strain Belgium/Etalle/wb/2018: complete genomic sequence and comparative analysis with reference p72 genotype II strains. Transbound Emerg Dis. 2019. 10.1111/tbed.13302. 10.1111/tbed.13302 31332955

[8] Wang A, Jia R, Liu Y, Zhou J, Qi Y, Chen Y, et al. Development of a novel quantitative real-time PCR assay with lyophilized powder reagent to detect African swine fever virus in blood samples of domestic pigs in China. Transbound Emerg Dis. 2019. 10.1111/tbed.13350. 10.1111/tbed.13350 31483566

[9] Wozniakowski G, Kozak E, Kowalczyk A, Lyjak M, Pomorska-Mol M, Niemczuk K (2016). Current status of African swine fever virus in a population of wild boar in eastern Poland (2014-2015). Arch Virol.

[10] Chapman DA, Darby AC, Da Silva M, Upton C, Radford AD, Dixon LK. Genomic analysis of highly virulent Georgia 2007/1 isolate of African swine fever virus. Emerg Infect Dis 2011;17(4):599–605; doi: 10.3201/eid1704.101283. 10.3201/eid1704.101283 PMC3379899 21470447

[11] Ge S, Li J, Fan X, Liu F, Li L, Wang Q (2018). Molecular characterization of African swine fever virus, China, 2018. Emerg Infect Dis.

[12] Gallardo C, Fernández-Pinero J, Arias M. African swine fever (ASF) diagnosis, an essential tool in the epidemiological investigation. Virus Res. 2019;271:197676; doi: https://doi.org/10.1016/j.virusres.2019.197676. 10.1016/j.virusres.2019.197676 31362027

[13] Petrovan V, Yuan F, Li Y, Shang P, Murgia MV, Misra S (2019). Development and characterization of monoclonal antibodies against p30 protein of African swine fever virus. Virus Res.

[14] Gallardo C, Nieto R, Soler A, Pelayo V, Fernandez-Pinero J, Markowska-Daniel I (2015). Assessment of African swine fever diagnostic techniques as a response to the epidemic outbreaks in eastern European Union countries: how to improve surveillance and control programs. J Clin Microbiol.

[15] East-Seletsky A, O'Connell MR, Knight SC, Burstein D, Cate JH, Tjian R (2016). Two distinct RNase activities of CRISPR-C2c2 enable guide-RNA processing and RNA detection. Nature..

[16] Gootenberg JS, Abudayyeh OO, Lee JW, Essletzbichler P, Dy AJ, Joung J (2017). Nucleic acid detection with CRISPR-Cas13a/C2c2. Science..

[17] Li SY, Cheng QX, Liu JK, Nie XQ, Zhao GP, Wang J (2018). CRISPR-Cas12a has both cis- and trans-cleavage activities on single-stranded DNA. Cell Res.

[18] Teng F, Guo L, Cui T, Wang XG, Xu K, Gao Q (2019). CDetection: CRISPR-Cas12b-based DNA detection with sub-attomolar sensitivity and single-base specificity. Genome Biol.

[19] Myhrvold C, Freije CA, Gootenberg JS, Abudayyeh OO, Metsky HC, Durbin AF (2018). Field-deployable viral diagnostics using CRISPR-Cas13. Science..

[20] Gootenberg JS, Abudayyeh OO, Kellner MJ, Joung J, Collins JJ, Zhang F (2018). Multiplexed and portable nucleic acid detection platform with Cas13, Cas12a, and Csm6. Science..

[21] Chen JS, Ma E, Harrington LB, Da Costa M, Tian X, Palefsky JM (2018). CRISPR-Cas12a target binding unleashes indiscriminate single-stranded DNase activity. Science..

[22] Sashital DG (2018). Pathogen detection in the CRISPR-Cas era. Genome Med.

[23] King DP, Reid SM, Hutchings GH, Grierson SS, Wilkinson PJ, Dixon LK (2003). Development of a TaqMan PCR assay with internal amplification control for the detection of African swine fever virus. J Virol Methods.

[24] Fonfara I, Richter H, Bratovic M, Le Rhun A, Charpentier E. The CRISPR-associated DNA-cleaving enzyme Cpf1 also processes precursor CRISPR RNA. Nature. 2016;532(7600):517–521; doi: 10.1038/nature17945. 10.1038/nature17945 27096362

[25] Miao F, Zhang J, Li N, Chen T, Wang L, Zhang F (2019). Rapid and sensitive recombinase polymerase amplification combined with lateral flow strip for detecting African swine fever virus. Front Microbiol.

[26] Bai J, Lin H, Li H, Zhou Y, Liu J, Zhong G (2019). Cas12a-based on-site and rapid nucleic acid detection of African swine fever. Front Microbiol.

[27] Wang X, Ji P, Fan H, Dang L, Wan W, Liu S (2020). CRISPR/Cas12a technology combined with immunochromatographic strips for portable detection of African swine fever virus. Commun Biol.

[28] Li SY, Cheng QX, Wang JM, Li XY, Zhang ZL, Gao S (2018). CRISPR-Cas12a-assisted nucleic acid detection. Cell Discov.

[29] Fernandez-Pinero J, Gallardo C, Elizalde M, Robles A, Gomez C, Bishop R (2013). Molecular diagnosis of African swine fever by a new real-time PCR using universal probe library. Transbound Emerg Dis.

[30] Liu L, Luo Y, Accensi F, Ganges L, Rodriguez F, Shan H (2017). Pre-clinical evaluation of a real-time PCR assay on a portable instrument as a possible field diagnostic tool: experiences from the testing of clinical samples for African and classical swine fever viruses. Transbound Emerg Dis.

[31] Niederwerder MC, Stoian AMM, Rowland RRR, Dritz SS, Petrovan V, Constance LA (2019). Infectious dose of African swine fever virus when consumed naturally in liquid or feed. Emerg Infect Dis.

