# Supplementary material for: Development and clinical application of a novel CRISPR-Cas12a based assay for the detection of African swine fever virus
Source: BMC Microbiol. 2020 Sep 14;20:282. doi: 10.1186/s12866-020-01966-6 (PMC7491166; doi:10.1186/s12866-020-01966-6)
Supplement: Supplementary file 1 — Additional file 1: Table S1. List of crRNA spacer sequences and DNA reporters used in this study. Table S2. List of RPA and conventional PCR primer sequences used in this study. Table S3. The sensitivity comparison between commercial qPCR kit and OIE-recommended qPCR. Table S4. The specificity comparison between commercial qPCR kit and OIE-recommended qPCR [file 12866_2020_1966_MOESM1_ESM.docx]

Development and clinical application of a novel CRISPR-Cas12a based assay for the detection of African swine fever virus

Xiaoying Wang^1§^, Sheng He^1§^, Na Zhao^2^, Xiaohong Liu^1^, Yongchang Cao^1^, Guihong Zhang^3, 4^, Gang Wang^2*^, Chunhe Guo^1*^

^1^State Key Laboratory of Biocontrol, School of Life Sciences, Sun Yat-sen University, North Third Road, Guangzhou Higher Education Mega Center, Guangzhou, Guangdong 510006, PR China

^2^Precise Genome Engineering Center, School of Life Sciences, Guangzhou University, Guangzhou, Guangdong 510006, PR China

^3^College of Veterinary Medicine, South China Agricultural University, Guangzhou, Guangdong 510642, PR China

^4^African Swine Fever Regional Laboratory of China (Guangzhou), Guangzhou, Guangdong 510642, PR China.

^§^These authors contributed equally to this work.

^*^Correspondence: [guochunh@mail.sysu.edu.cn](mailto:guochunh@mail.sysu.edu.cn) (C.G.); [wanggang.v@gzhu.edu.cn](mailto:wanggang.v@gzhu.edu.cn) (G.W.)

Supplementary Material

Supplementary Table S1 List of crRNA spacer sequences and DNA reporters used in this study.

| crRNA Name | Sequence (5'-3') |
| --- | --- |
| crRNA1 | AAUCCUAUAAACAUAUAUUC |
| crRNA2 | AGAGCAGACAUUAGUUUUUCAUCG |
| crRNA3 | AGGGGUUACAAACAGGUUAUUG |
| crRNA4 | GCGAUGCAAGCUUUAUGGUG |
| crRNA5 | CGAUGCAAGCUUUAUGGUGAUAAA |
| crRNA6 | UGGUGAUAAAGCGCUCGCCG |
| crRNA7 | GGGGUUUGAGGUCCAUUACAGCU |
| crRNA8 | CCUGCUGUUUGGAUAUU |
| crRNA9 | CAUCGGUAAGAAUAGGUUUGCU |
| crRNA10 | UUCGAUUUGACUCAAAGUGGGUUC |
| crRNA11 | CAUCAAAGUUCUGCAGCUCUUACA |
| crRNA12 | AGGAUAGAGAUACAGCUCUUCCAG |
| crRNA13 | CAUCAAUAACCUGUUUGUAACCCC |
| crRNA14 | AACCAUGGUUUAUCCCAGGAGU |
| crRNA15 | UCACCAUAAAGCUUGCAUCGCAAA |
| crRNA16 | AGUCAAAUCGAAGAAACACAU |
| crRNA17 | UCUUAUUGCUAACGAUGGGAAG |
| crRNA18 | CAUCAAAGUUCUGCAGC |
| crRNA19 | UCGAUAAGAUUGAUACC |
| HEX-N6-BHQ1 | HEX-NNNNNN-BHQ1 |

Supplementary Table S2 List of RPA and conventional PCR primer sequences used in this study

| Primers | Oligonucleotides (5'-3') | Corresponding to crRNA |
| --- | --- | --- |
| RPA1-F | ATATGACCACTGGGTTGGTATTCCTCCCGT | crRNA11, 12, 18, 19 |
| RPA1-R | ATCAACACCGAGATTGGCACAAGTTCGGAC |  |
| RPA2-F | GTTAACAACATGTCCGAACTTGTGCCAATC | crRNA1, 2, 3, 4, 5, 6, 13, 14, 15 |
| RPA2-R | GAGAACGTGAACCTTGCTATTCCCTCAGTA |  |
| RPA3-F | GGAATAGCAAGGTTCACGTTCTCATTAAAC | crRNA7, 8, 9 |
| RPA3-R | AGTGGCCCTCTCCTATGCAACATTCATGAT |  |
| RPA4-F | CCTGAATCGGAGCATCCTGCCAGGATGAAT | crRNA10, 16, 17 |
| RPA4-R | ATGGCATCAGGAGGAGCTTTTTGTCTTATT |  |
| PCR-F | GTACTGTAACGCAGCACAG |  |
| PCR-R | GGCACAAGTTCGGACATGT |  |

Supplementary Table S3 The sensitivity comparison between commercial qPCR kit and OIE-recommended qPCR

| Copy number  (copies/μl) | Commercial qPCR kit  (Ct value) | | | OIE-recommended qPCR  (Ct value) | | |
| --- | --- | --- | --- | --- | --- | --- |
|  | 1^a^ | 2 | 3 | 1 | 2 | 3 |
| 580 | 28.78 | 28.66 | 28.69 | 29.04 | 29.17 | 28.73 |
| 116 | 30.66 | 30.52 | 30.55 | 31.14 | 31.19 | 31.26 |
| 11.6 | 33.94 | 33.97 | 34.05 | 34.49 | 34.65 | 34.96 |
| 1.16 | - | - | - | - | - | - |
| 0.116 | - | - | - | - | - | - |

^a^ n = 3 technical replicates.

Supplementary Table S4 The specificity comparison between commercial qPCR kit and OIE-recommended qPCR

| Swine viruses | Commercial qPCR kit  (Ct value) | | | OIE-recommended qPCR  (Ct value) | | |
| --- | --- | --- | --- | --- | --- | --- |
|  | 1^a^ | 2 | 3 | 1 | 2 | 3 |
| ASFV | 28.23 | 28.15 | 29.11 | 29.32 | 29.36 | 28.51 |
| PRRSV | - | - | - | - | - | - |
| CSFV | - | - | - | - | - | - |
| PCV2 | - | - | - | - | - | - |
| PRV | - | - | - | - | - | - |
| PEDV | - | - | - | - | - | - |
| TGEV | - | - | - | - | - | - |
| PPV | - | - | - | - | - | - |
| JEV | - | - | - | - | - | - |

^a^ n = 3 technical replicates.
